# Computational Prediction of the Complete Adsorption–Regeneration Cycle of Functionalized Metal–Organic Frameworks for Atmospheric Water Harvesting

**DOI:** 10.1021/acsanm.5c04879

**Published:** 2026-01-20

**Authors:** Mehrzad Arjmandi, Mohamed Khayet

**Affiliations:** † Department of Structure of Matter, Thermal Physics and Electronics, Faculty of Physics, 16734 University Complutense of Madrid, Avda. Complutense s/n, 28040 Madrid, Spain; ‡ Madrid Institute for Advanced Studies of Water (IMDEA Water Institute), Avda. Punto Com N◦ 2, 28805 Alcalá de Henares, 28040 Madrid, Spain

**Keywords:** metal docking, metal organic framework, atmospheric water harvesting, density functional theory, grand canonical Monte Carlo, kinetic Monte Carlo, molecular dynamics

## Abstract

This work presents a comprehensive multiscale computational investigation of the complete adsorption–regeneration cycle of functionalized adsorbents for atmospheric water harvesting (AWH) under low-humidity conditions. The target system is copper-docked MOF-303, a nanoporous framework with well-defined nanoscale channels and adsorption sites, functionalized with Amino and Nitro clusters, selected to tailor host–guest interactions and optimize water uptake and release. The simulations capture key molecular-level phenomena including host–guest interactions, water mobility, adsorption kinetics, and regeneration temperatures, providing a detailed picture of performance under varying environmental conditions. Cu–NH_2_@MOF-303 showed the highest water capacity, reaching a ∼38% increase over the pristine structure, while Cu–NO_2_@MOF-303 achieves an improvement of ∼25%. The kinetics follow a similar trend: the pristine framework saturates in 4 min at 2000 Pa, whereas Cu–NH_2_@MOF-303 reaches equilibrium almost instantaneously (∼0.1 min). Cu–NO_2_@MOF-303 also accelerates uptake, saturating within <3 min at 2000 Pa. Density functional theory (DFT) results confirm the enhanced affinity, with adsorption energies shifting from −84.77 kJ mol^–1^ in pristine MOF-303 to −99.93 kJ mol^–1^ (Cu–NH_2_) and −90.16 kJ mol^–1^ (Cu–NO_2_). Despite requiring a modestly higher regeneration temperature (≈25 K above pristine MOF-303), Cu–NH_2_@MOF-303 offers a favorable balance between stronger binding and practical desorption, making it suitable for low-to-moderate relative humidity conditions.

## Graphical abstract

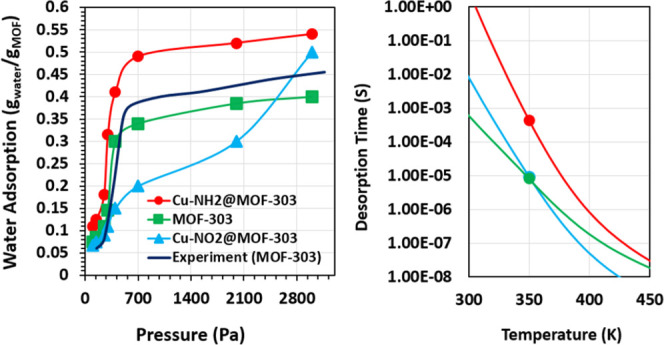

## 1 Introduction

Solar-driven sorption-based atmospheric water harvesting (SAWH) has emerged as a promising approach to address the increasing demand for clean water worldwide.^1^ These systems typically work through a two-stage process. First, water vapor is captured by a nanoporous material that captures moisture from arid air. In the second stage, the material is heated by solar irradiation, causing the water to be released as concentrated vapor, which can then be condensed into liquid water for collection. For a sorbent to be effective in such a system, it must rapidly capture water vapor from the air at low relative humidity (RH) levels, while also allowing for its release without requiring excessive energy.^2^

Metal–organic frameworks (MOFs), composed of metal ions or clusters coordinated with organic ligands, have emerged as outstanding adsorbents for SAWH owing to their exceptionally high surface area, tunable porosity, and versatile chemical functionality.^2,3^ Their highly porous structures offer abundant adsorption sites for water molecules, and their water sorption behavior can be precisely engineered to span from strongly hydrophilic to hydrophobic,^4–6^ enabling them to mimic the performance of a broad spectrum of sorbents such as aluminosilicates, zeolites, and nonpolar polymeric materials.^7–9^ One of the leading materials in this field is MOF-303, which is a high-performance MOF composed of rod-shaped aluminum-based secondary building units (SBUs) connected by 1-*H*-pyrazole-3,5-dicarboxylate (PZDC) linkers.^10^ This structure forms one-dimensional hydrophilic nanoscale channels approximately 6 Å wide, allowing for rapid water diffusion and high uptake, reaching up to 0.48 g g^–1^ under 20–40% relative humidity at 30 °C.^11–13^ The material exhibits excellent hydrolytic stability over more than 150 adsorption–desorption cycles and has demonstrated practical performance in water harvesting devices, yielding up to 1.3 L kg^–1^ day^–1^ indoors (32% RH, 27 °C) and 0.7 L kg^–1^ day^–1^ in arid climates such as the Mojave Desert (10% RH, 27 °C).^13^

Recent progress in nanoscale engineering of adsorbents has enabled researchers to fine-tune their adsorption characteristics to match specific environmental and application needs.^14–20^ Among various modification strategies, postsynthetic functionalization via the incorporation of external metal ions has emerged as an effective approach. This method can enhance adsorption capacity, selectivity, and structural stability. These modifications are typically achieved either by coordinating metal ions to the framework’s SBUs or by anchoring them to the organic linkers, often using chelating groups such as bipyridyl or pyrazole-based moieties.^21^ MOF 303, constructed from aluminum oxygen rod-shaped SBUs and PZDC linkers, has proven to be a structurally stable and highly adaptable candidate for such modifications.^22^ The spatial orientation of its uncoordinated nitrogen atoms allows for the precise introduction of monovalent metal ions like Cu­(I) and Ag­(I), as demonstrated in previous postsynthetic metalation studies.^21^ These modified frameworks have shown improved performance in a variety of applications, including xenon gas separation, heavy metal removal, and the capture of radioactive iodine, all while maintaining structural integrity under challenging conditions such as humidity and elevated temperatures.^21,23,24^ For instance, a detailed structural investigation of Cu­(I)-metalated MOF-303 has revealed that the copper centers are coordinated by two nitrogen atoms from adjacent PZDC linkers, along with a third ligand identified as a chloride ion, forming a CuN_2_Cl coordination geometry.^21^ This well-defined arrangement confirms not only the chelation capability of the pyrazole-based linker system but also the capacity of the Cu­(I) center to accommodate an additional ligand beyond the two nitrogen donors. This structural feature opens the possibility of tailoring the nanoscale coordination environment of the metal site by substituting the chloride ligand with other functional groups.

Building on previous insights, this work investigates the incorporation of amino (NH_2_) and nitro (NO_2_) groups in place of chloride within the copper coordination sphere of MOF-303. The choice of these polar functional groups is motivated by their distinct electronic properties: NH_2_ acts as an electron-donating group, which can enhance metal–ligand interactions, modify the nanoscale electrostatic environment, and increase affinity for polar adsorbates, while NO_2_, as a strong electron-withdrawing substituent, can modify the electron density around the metal center, influencing coordination preferences and adsorption dynamics.^25–27^ Despite the promising potential of postsynthetic metal docking combined with such targeted functionalization, the effect of NH_2_ and NO_2_ incorporation on MOF-303’s water harvesting performance remains largely unexplored. We hypothesize that replacing the chloride ligand in Cu­(I)-MOF-303 with strongly electron-donating (NH_2_) and electron-withdrawing (NO_2_) groups will allow us to fine-tune the water adsorption energy, thereby optimizing the trade-off between high water uptake and low regeneration energy. Therefore, this study aims to elucidate how these chemical modifications impact the framework’s structural characteristics and water adsorption–desorption behavior. To achieve this, a comprehensive multiscale modeling approach is employed, including grand canonical Monte Carlo (GCMC) simulations for adsorption isotherms, kinetic Monte Carlo (KMC) for adsorption kinetics, density functional theory (DFT) for electronic structure analysis, and molecular dynamics (MD) for investigating water transport within nanoscale pores. This integrated methodology provides fundamental insights to guide the rational design of advanced MOFs tailored for efficient atmospheric water harvesting (AWH) applications.

## 2 Models and Methods

### 2.1 Models

A hybrid approach combining classical and quantum computational techniques was utilized in this research to analyze the structural features and adsorption behavior of both unmodified MOF-303 and its Cu–X functionalized derivatives (X: NH_2_, NO_2_). The crystallographic information for MOF-303 was obtained from the Cambridge crystallographic data centre (CCDC).^28^ A central segment extracted from a supercell consisting of four unit cells served as the simulation domain for MD, GCMC, and KMC simulations, as illustrated in Figure 1. For the electronic structure analysis, a simplified molecular model was created to reflect the local coordination environment, which was subsequently analyzed using DFT calculations (Figure 2a–e). All computational analyses were conducted for both the pristine MOF-303 and the Cu–X@MOF-303 (X: NH_2_, NO_2_) structures.

**Figure 1:**
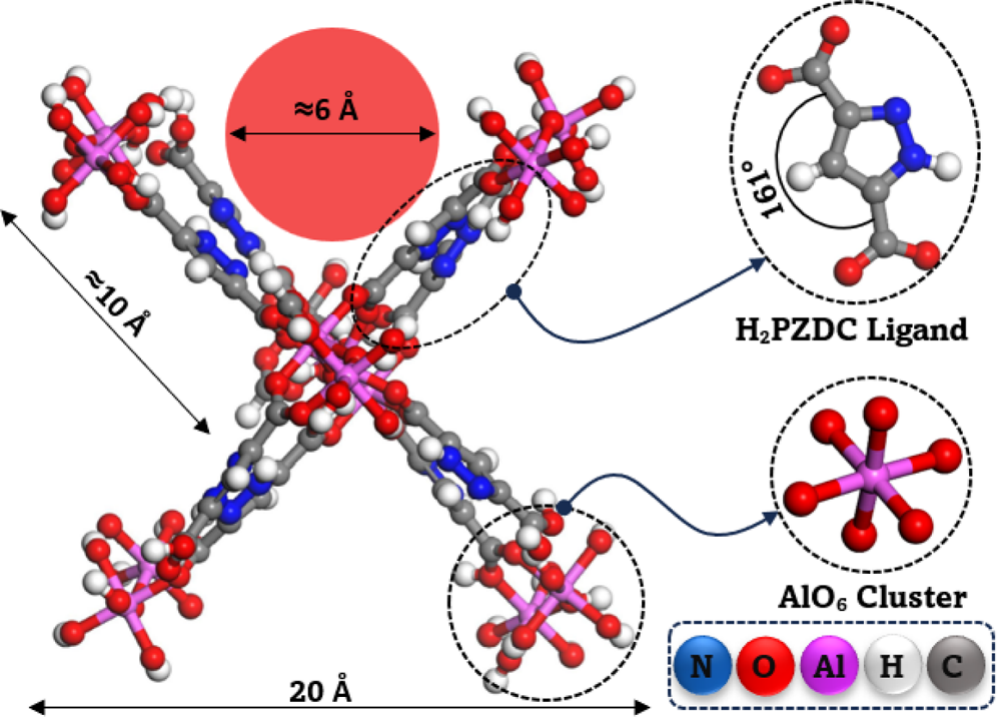
Structural model of MOF-303 used in the simulations, showing the pore size. For clarity, the ligand and metal cluster are displayed separately.

**Figure 2:**
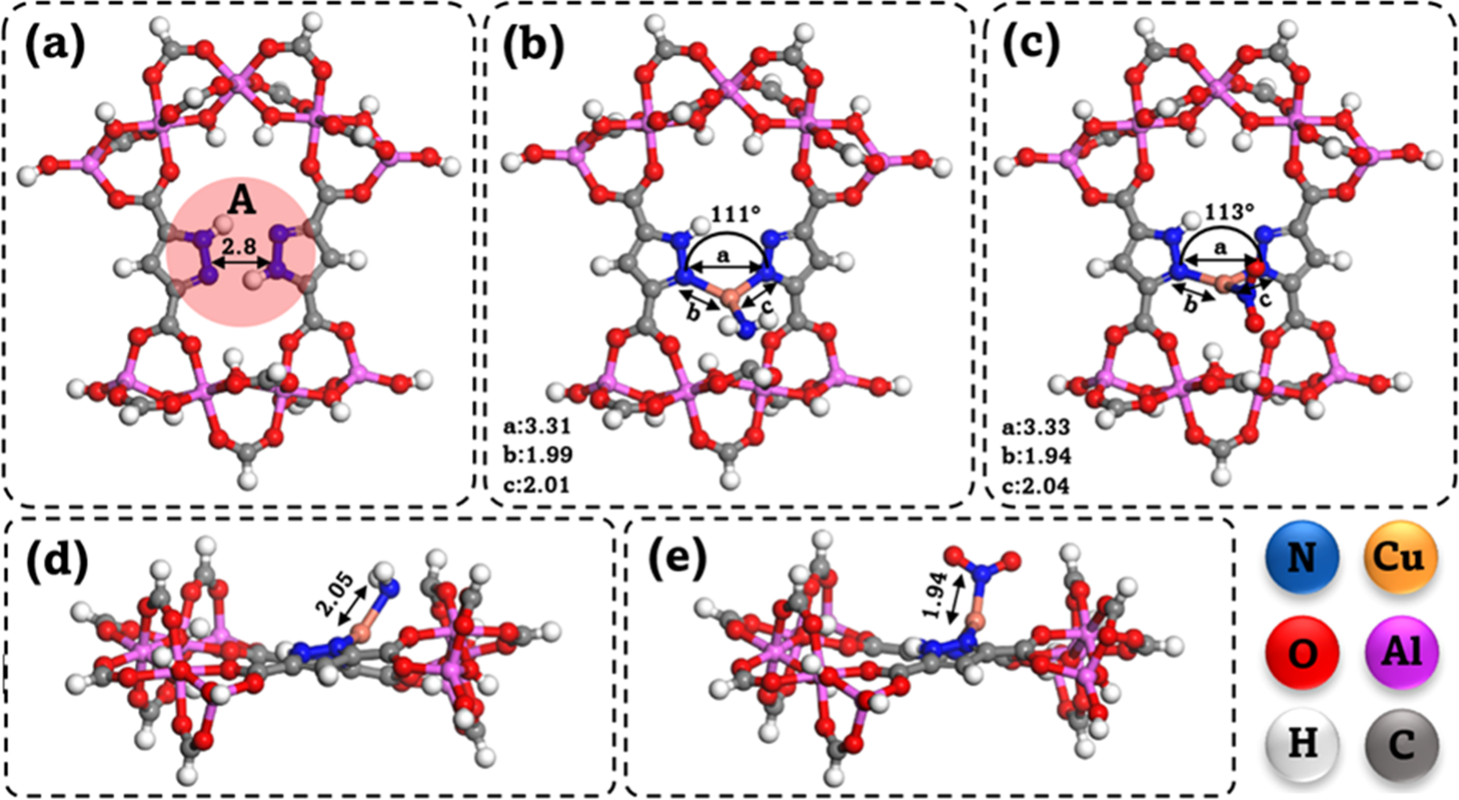
Simplified DFT models of (a) pristine MOF-303; (b,d) Cu–NH_2_@MOF-303; and (c,e) Cu–NO_2_@MOF-303. Bond angles and distances (in Å) are taken from the optimized geometries.

### 2.2 Ab Initio Calculations

To explore how water molecules interact with both unmodified and functionalized MOF-303 structures, a set of first-principles calculations based on DFT using the Gaussian 16W software package. The computations were executed at the B3LYP level of theory, utilizing a combination of SDD and LANL2DZ basis sets to maintain a balance between computational cost and accuracy, particularly suitable for MOF clusters incorporating transition metals.^29–32^ During geometry optimization, terminal atoms were constrained to their crystallographic positions to maintain the structural integrity of the framework, while the internal atoms were allowed to relax completely. After optimization, adsorption energies of water molecules were determined using single-point energy calculations according to the equation

1 $$E_{ads}=E_{MOF+water}-\left(E_{MOF}+E_{water}\right)$$

where *E*_MOF+water_ is the total energy of the water-MOF complex, and *E*_water_ and *E*_MOF_ represents the total energies of the isolated water and MOF molecule, respectively.^33,34^

In addition, a comprehensive set of electronic structure calculations was carried out to further elucidate the electronic characteristics of the studied systems. Molecular electrostatic potential (MEP) maps were employed to identify regions of electron-rich and electron-deficient character across the surfaces of both unmodified and Cu–X functionalized MOF-303 (X: NH_2_ and NO_2_) variants. Frontier molecular orbital analysis was performed to examine how functional groups (i.e., Cu–NH_2_ and Cu–NO_2_) and the presence of adsorbed water molecules influence the electronic distribution and reactive behavior of the frameworks. The energies and spatial configurations of the highest occupied molecular orbital (HOMO) and the lowest unoccupied molecular orbital (LUMO) were obtained within the conceptual DFT framework. These orbitals serve as key indicators of electronic activity, providing insights into parameters such as charge-transfer potential, electronic polarizability, and anisotropy in host–guest interactions. To delve deeper into electronic distribution patterns within the structures, natural bond orbital (NBO) and Mulliken population analyses were conducted to evaluate charge localization and delocalization effects introduced by Cu–NH_2_ and Cu–NO_2_ substitutions. Furthermore, the dipole moment (μ) was calculated for each optimized structure to quantify how functionalization impacts the overall molecular polarity.

### 2.3 Grand Canonical and Kinetic Monte Carlo Simulations

While RASPA^21^ is widely employed for Monte Carlo simulations in porous frameworks, this work utilized the LAMMPS software suite to establish a unified simulation environment capable of performing GCMC, KMC, and MD simulations within a single platform. The decision was motivated by the goal of capturing not only equilibrium adsorption characteristics but also the kinetic behavior and spatial effects resulting from Cu–NH_2_ and Cu–NO_2_ modifications. LAMMPS’s scripting flexibility supported the implementation of hybrid MC/MD protocols and residence-time-based kinetic algorithms. This multiscale setup enabled a comprehensive analysis of both thermodynamic and kinetic aspects of water sorption in both unmodified and doped MOF-303 systems, under conditions relevant to atmospheric water harvesting applications. An automated Python-based pipeline was developed to streamline simulations and prepare structural inputs across multiple scenarios. In GCMC simulations, water molecules were allowed to translate, rotate, be reinserted, or swapped, while framework atoms remained fixed.

GCMC simulations were performed at three temperatures (300, 308, and 318 K) to capture temperature-dependent adsorption behavior representative of practical SAWH operating conditions. Water molecules were randomly inserted into the accessible pore volume of the MOF frameworks, ensuring that no overlaps occurred with the host atoms. Insertion and deletion moves were accepted or rejected using the Metropolis criterion, based on the chemical potential corresponding to the target pressure and temperature. Translation, rotation, swap, and reinsertion moves were included to achieve thorough sampling of the configurational space, capturing both clustering behavior and site-specific adsorption. During reinsertion moves, water molecules were randomly repositioned within the pore space, with repeated attempts made whenever initial placements overlapped with the framework atoms. To apply the desired RH conditions, the corresponding water vapor pressures were first determined from the saturation vapor pressure at the simulation temperature. These vapor pressures were then converted to the chemical potentials used by the GCMC algorithm to regulate insertion and deletion attempts. In other words, RH was incorporated indirectly through its associated equilibrium vapor pressure, which defined the thermodynamic driving force for adsorption in the simulation. Each system was equilibrated for 1 × 10^6^ Monte Carlo steps, followed by 5 × 10^6^ production steps, yielding more than 10,000 distinct water configurations for analysis. The convergence of simulations was monitored by tracking the stability of the average number of adsorbed water molecules and event rates over the production steps, confirming that sufficient sampling was achieved for statistically meaningful results. The average number of water molecules adsorbed in the pores at a given vapor pressure or RH was obtained directly from the GCMC simulations. This equilibrium loading was then used as input for the subsequent MD simulations. The bulk density of water within the pores was calculated as the total mass of adsorbed water divided by the accessible pore volume of the MOF, providing a measure of local water content under the given RH conditions. The accessible pore volume of the MOF was determined from the crystal structure using a probe-based geometric analysis, representing the volume available for water molecules within the pores.

Intermolecular forces were calculated using a combination of Lennard–Jones (12–6) and electrostatic interactions. Specific computational parameters are detailed in the Supporting Information (eqs S1–S3).^10,35^ The Lorentz–Berthelot mixing rules were employed for cross-interactions between dissimilar atoms. A 12 Å cutoff was used for LJ potentials, and long-range electrostatics were computed using the Ewald summation method with a precision of 10^–6^. The DREIDING^36^ and universal force field (UFF)^37^ force fields provided the interaction parameters for the MOF systems. The specific values of ε and σ for key atoms used in our simulations can be found in Table 1.^36,37^ Atomic charges were assigned using the charge equilibration method, informed by DFT-based NBO calculations in Gaussian. NBO charges are considered to provide a more physically meaningful representation of the electron density compared to Mulliken charges, as they are less sensitive to molecular geometry or basis set choice and capture polarization effects in atoms and bonds more accurately. These characteristics make NBO charges reasonable for systems containing polar functional groups or strong electrostatic fields, such as the MOFs used in this study. This approach has been widely applied in previous studies of materials with strong electrostatic fields and polar functional groups,^38–42^ showing reliable results. The SPC/E model^43^ was used to represent water molecules.

**Table 1:** Parameters (ε, σ) of the Lennard–Jones Potential Function for the Key Atoms in This Study

element	σ (Å)	ε (kcal mol^−1^)
Al	4.50	0.505
Cu	3.49	0.005
N	3.66	0.077
H	3.19	0.015
O	3.41	0.096
C	3.89	0.095

To investigate the time-dependent behavior of water sorption, KMC simulations were employed to model adsorption, desorption, and diffusion events at a molecular level.^44,45^ The simulations used the Bortz–Kalos–Lebowitz (BKL) residence-time algorithm,^46,47^ which is particularly well suited for systems dominated by rare, thermally activated events. In this framework, the mobile species were individual water molecules or small water clusters occupying discrete adsorption sites within the rigid MOF lattice. The time associated with each event is dynamically determined based on the rates of all possible events. This means there is no fixed time step; faster events contribute to smaller increments of time, while rarer events contribute to larger increments. Three classes of elementary events were considered: (i) adsorption into an available site, (ii) desorption from an occupied site, and (iii) diffusion (hopping) between neighboring sites. The MOF framework was treated as rigid, and no bulk liquid water phase was present; thus, all transitions occurred along predefined site-to-site pathways rather than continuous atomic trajectories.

For adsorption, the adsorption sites and their energies were determined using DFT calculations, yielding a detailed map of water adsorption sites within the MOF. For all adsorption events, we applied the nudged elastic band (NEB) method^47–49^ to identify the minimum-energy pathways and the associated saddle points. The NEB procedure involves generating intermediate “images” connecting the initial and final configurations, where the energy of each image is relaxed to map the reaction pathway. The highest-energy image in the NEB method defines the activation barrier for adsorption (*E*_ads_). For desorption, the desorption energy is approximated by taking the absolute value of the adsorption energy (*E*_ads_) as the desorption barrier (*E*_des_). This simplification assumes that the desorption process follows a reverse path with the same energy magnitude. For diffusion, the diffusion events (hopping between neighboring sites) were analyzed using the NEB method as well. The energy for diffusion barriers (*E*_diff_) was calculated by identifying the transition state between two adjacent adsorption sites. Again, a series of intermediate images was generated, and the energy of each image was relaxed to map the diffusion process. The highest-energy image in this case defines the activation barrier for diffusion. These calculations were performed using the VASP software, and harmonic vibrational corrections were applied to obtain temperature-dependent free-energy barriers. This combination of DFT adsorption energies and NEB-derived barriers allowed the construction of a consistent kinetic landscape for water transport within the MOF.

The barrier energy *E*_*i*_ obtained from the highest-energy NEB image is used to compute the rate constant *k*_*i*_ for simple diffusion and reaction processes, which for processes on a surface can be approximate as^50^

2 $$k_{i}=A_{i}.\exp\left(\frac{-{\;E}_{i}}{RT}\right)$$

where *A*_*i*_ is the pre-exponential factor, *R* is the gas constant, and *T* is the absolute temperature. KMC simulations were performed at 300 K, matching the lower-bound operating temperature commonly examined in SAWH studies and ensuring consistency with equilibrium GCMC inputs. This KMC framework enabled efficient sampling of rare processes and offered a more accurate depiction of water transport and uptake kinetics within the MOF structures.

### 2.4 Molecular Dynamic Simulations

To analyze the real-time motion of water molecules within pristine and Cu–X functionalized MOF-303 (X: NH_2_ and NO_2_) structures, molecular dynamics (MD) simulations were conducted under ambient conditions using the LAMMPS simulation package. The simulations were performed in the canonical (NVT) ensemble, keeping the framework atoms fixed, while allowing full flexibility for the internal motion of water molecules. All MD simulations were carried out at a constant temperature of 300 K, maintained using the Nosé–Hoover thermostat, consistent with the temperature conditions implemented in the KMC simulations.^51^ The simulation box had dimensions of 28 × 28 × 28 Å^3^ under periodic boundary conditions, and a time step of 2.0 fs was used throughout the MD simulations. All force field parameters and interaction potentials were consistent with those described in Section 2.3. A fully automated pipeline built with Python facilitated structure setup, trajectory generation, and analysis, with MD Analysis used to extract structural and dynamic descriptors.

To investigate local interactions between water and functional groups, radial distribution functions (RDFs) were computed between selected atomic pairs, including oxygen in water (OW) and hydrogen in –NH_2_, and hydrogen in water (HW) and oxygen in –NO_2_ groups. These calculations were carried out for both unmodified and functionalized MOF-303 structures under two water vapor pressures (250 and 700 Pa), representing pre- and poststep regions of the adsorption isotherm. Additional computational specifics are available in Supporting Information (eq. S4).^52,53^

To further explore water mobility under confinement, the mean square displacement (MSD) of water molecules was evaluated, providing insight into translational dynamics across different MOF structures. The corresponding self-diffusion coefficients (*D*) were derived from MSD data using Einstein’s relation, with methodological details given in eqs. S5 and S6 of the Supporting Information.^54,55^

For MD simulations, the number of water molecules was fixed at the equilibrium loading obtained from GCMC, ensuring that the dynamic properties (RDF, MSD) reflect the same thermodynamic adsorption conditions. During the simulations, a total production run of 2500 ps was performed at 300 K, which provided sufficient sampling for both structural and dynamic analyses. RDFs were computed from the full trajectory to characterize local interactions between water molecules and functional groups, while the MSD was evaluated over the same trajectory to extract translational diffusion coefficients. The simulation length ensured convergence of both RDF and MSD analyses under the equilibrium loading obtained from GCMC simulations. Convergence of MSD and RDF analyses was confirmed by monitoring the stability of these properties over sequential segments of the trajectory, ensuring that the computed averages were statistically meaningful. For MSD calculations, the displacement of each water molecule is measured relative to its initial position at the start of the MD simulation, corresponding to the adsorption site highlighted as region A in Figure 2a.

### 2.5 Temperature-Dependent Desorption Behavior

MOF-303 is a microporous and structurally rigid sorbent that exhibits minimal to no hysteresis in its water sorption behavior, as reported in previous experimental studies.^13^ At the same time, pristine and especially functionalized variants of MOF-303 are characterized by high adsorption strength toward water,^56^ reflecting strong interactions between water molecules and the framework, particularly at primary binding sites. High adsorption strength at primary binding sites related to higher energy barriers for desorption.^57^ In standard GCMC simulations, when energy barriers are sufficiently high relative to the simulation time scale, the system may become trapped in metastable states, leading to stepwise transitions and artificially observed hysteresis.^58^ Notably, this simulation-induced hysteresis can appear even for materials that experimentally exhibit reversible adsorption, as the actual energy barriers at experimental conditions may exceed those accessible within the finite simulation time.^58^ Therefore, for systems like MOF-303 with minimal experimental hysteresis but strong adsorption interactions, standard GCMC may not reliably reproduce the true desorption behavior. To overcome this limitation, other computational approaches are often combined with GCMC56,^58–60^ although this increases the computational cost and complexity.

Therefore, in this study desorption was evaluated using temperature-corrected DFT adsorption energies combined with transition state theory (TST), providing a physically rigorous and quantitatively reliable description of water release. In this study, the thermal contributions were computed using scaled DFT vibrational frequencies within the harmonic approximation,^61–63^ while translational and rotational contributions were treated using classical approximations.^63^ These approximations provide a practical way to include the main effects of molecular motion on desorption energies and kinetics at typical temperatures, yielding a temperature-dependent adsorption energy *E*_ads_ (*T*) that naturally accounts for enhanced molecular mobility and reduced binding strength at elevated temperatures. It is important to emphasize that this approach is an approximation. Translational and rotational contributions were treated classically, neglecting quantum effects, while vibrational contributions were evaluated using scaled DFT harmonic frequencies. Possible anharmonicities and other higher-order quantum effects are not considered, and thus the thermal corrections may slightly over- or underestimate the true values in some cases. Despite these limitations, this approach captures the dominant trends and provides a computationally efficient way to incorporate temperature effects, sufficient for estimating regeneration temperatures and desorption times within the complete SAWH cycle. Detailed technical descriptions and the specific formulas used are provided in the Supporting Information (eqs. S7–S9). Using transition state theory (TST), the corresponding desorption time constant, τ­(*T*), was computed based on the Polanyi–Wigner equation^64–67^

3 $$\tau \left(T\right)=\vartheta_{0}^{-1}.\exp\left(\frac{-E_{ads}\left(T\right)}{K_{B}T}\right)$$

Where *E*_ads_ (*T*) is the temperature-dependent water adsorption energies, *T* is the temperature, and *K*_B_ is the Boltzmann constant. Here, ν_0_ denotes the attempt frequency, which represents the vibrational frequency of a water molecule in its adsorption site. It was estimated from the transition-state theory approximation *v*_0_ = *k*_B_/*h* (where *h* is Planck’s constant), giving values on the order of 10^12^–10^13^ s^–1^ within the investigated temperature range.^68–71^ Because its temperature variation is small compared to the exponential Arrhenius dependence, in this study ν_0_ was assumed to be a constant value of 6 × 10^12^ s^–1^. Increasing temperature enhances molecular motion, weakening adsorption and accelerating desorption kinetics.

## 3 Results and Discussion

### 3.1 Water Adsorption Study

Figure 3 illustrates the water adsorption isotherms obtained from GCMC simulations for pristine MOF-303 and its functionalized analogues Cu–X@MOF-303 (X = NH_2_, NO_2_). Figure 3a,b present the adsorption behavior at 300 K, while Figure 3c–e extend this analysis by showing temperature-dependent isotherms at 300, 308, and 318 K for each structure individually. For validation purposes, the simulated results are compared with experimental adsorption data for pristine MOF-303 measured at 298.15 K, taken from ref 72. As depicted in Figure 3a, the simulated isotherm for MOF-303 captures the key qualitative features observed experimentally, including a gradual increase in uptake at lower pressures followed by a sharp rise approaching saturation. The slightly lower absolute uptake in simulations is likely due to simplifications in the structural model. Functionalization with Cu–X units enhances total water uptake, with Cu–NH_2_@MOF-303 achieving the highest saturated capacity-approximately 38% greater than that of the unmodified MOF-303. Cu–NO_2_@MOF-303 also shows a significant improvement, with an increase of around 25%. This enhancement is consistent with the expected increase in hydrophilicity and the presence of additional adsorption sites introduced by functional groups.^25^ In contrast, Figure 3b, focusing on the low-pressure region (*P* ≤ 450 Pa), reveals a different trend. In this range, pristine MOF-303 adsorbs more water than Cu–NO_2_@MOF-303, despite its lower saturated capacity. Notably, Cu–NH_2_@MOF-303 maintains superior uptake even at low pressures, outperforming both the unmodified and NO_2_-functionalized variants. This behavior at low pressure can be rationalized by considering the competing effects of surface area reduction and increased hydrophilicity upon functionalization.^26^ While the introduction of –NO_2_ groups enhances total water uptake at high pressures due to increased polarity and additional binding sites, it may simultaneously reduce accessible surface area or alter pore environment in a way that is less favorable for initial water adsorption at low pressure. In contrast, the –NH_2_ functional group appears to provide favorable hydrogen bonding interactions, facilitating water uptake even in the low-pressure regime.^25^ Therefore, the observed differences in water adsorption behavior at low pressures among pristine MOF-303, Cu–NO_2_@MOF-303, and Cu–NH_2_@MOF-303 can be attributed to the balance between enhanced hydrophilicity and potential surface or structural limitations introduced by different functional groups.

**Figure 3:**
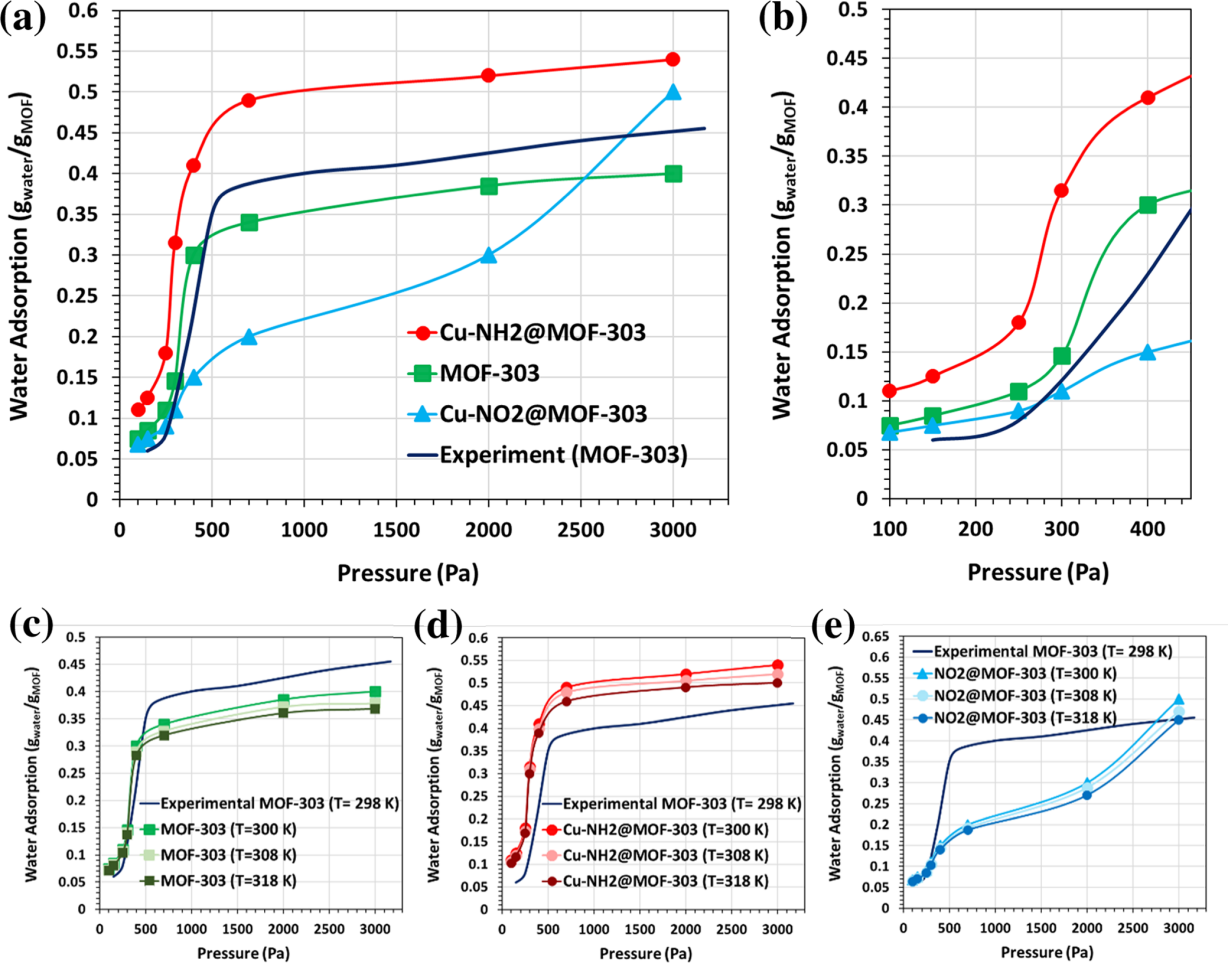
GCMC water adsorption isotherms for pristine MOF-303 and Cu–X@MOF-303 (X = NH_2_, NO_2_) at three temperatures (300, 308, and 318 K). Panels (a) and (b) show the full-range and low-pressure (*P* ≤ 450 Pa) isotherms at 300 K, including comparison of pristine MOF-303 with experimental data reported at 298.15 K for validation purposes.^72^ Panels (c–e) present the temperature-dependent isotherms for pristine MOF-303, Cu–NH_2_@MOF-303, and Cu–NO_2_@MOF-303, respectively.

The results highlight the dual effect of functional group type on water adsorption behavior. Electron-withdrawing groups like –NO_2_ enhance high-pressure uptake by increasing overall hydrophilicity and introducing additional binding sites but may limit low-pressure adsorption due to reduced accessible surface area or weaker initial interactions with water. In contrast, the –NH_2_ group improves both low- and high-pressure performance by enabling stronger hydrogen bonding and maintaining favorable pore accessibility.

The additional temperature-dependent isotherms in Figure 3c–e demonstrate that increasing temperature systematically decreases water uptake for all adsorbents, in agreement with the exothermic nature of adsorption. This trend is particularly pronounced at higher pressures, where the adsorption mechanism transitions from initial site-filling to cooperative pore condensation. While all three structures exhibit this temperature sensitivity, Cu–NH_2_@MOF-303 consistently maintains the highest water uptake at each temperature, reaffirming the strong interaction between water and the amino-functionalized copper site.

The water adsorption kinetics of pristine and Cu–X (X = NH_2_, NO_2_) functionalized MOF-303 structures were evaluated under two representative pressures (700 and 2000 Pa) using KMC simulations (Figure 4a–c). For the pristine MOF-303 (Figure 4a), saturation was reached in approximately 9 min at ∼22% RH and 4 min at ∼63% RH, in good agreement with previous experimental findings,^13^ where adsorption times of ∼10 min at ∼20% RH and ∼2.5 min at ∼40% RH were reported. This consistency supports the reliability of the kinetic model and the employed force field. All systems exhibit faster uptake rates at higher pressure, consistent with the expected pressure dependence of adsorption kinetics.

**Figure 4:**
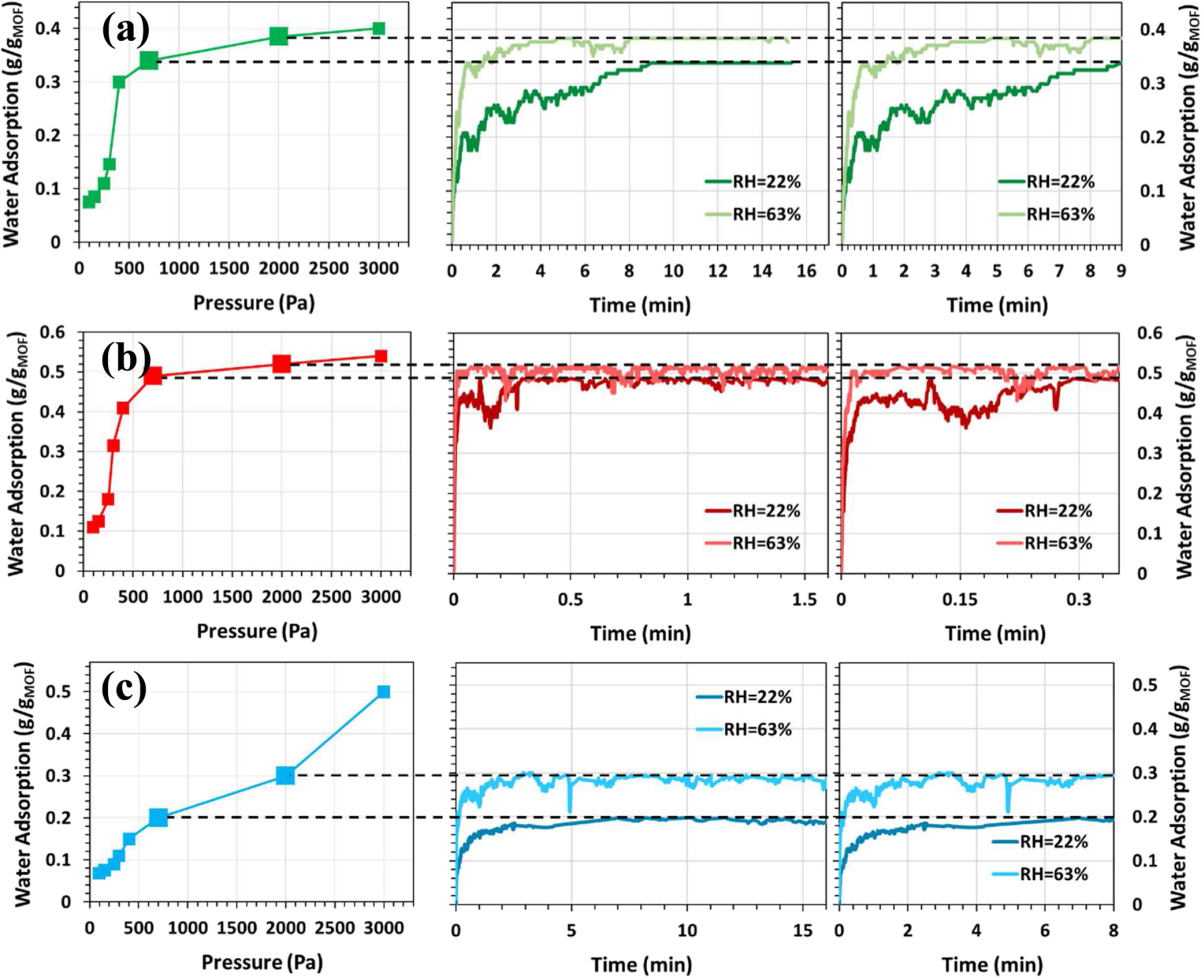
Water adsorption kinetics for (a) MOF-303, (b) Cu–NH_2_@MOF-303, (c) Cu–NO_2_@MOF-303 at 700 and 2000 Pa.

Functionalization with Cu–NH_2_ and Cu–NO_2_ species enhanced water uptake kinetics across all conditions. Cu–NH_2_@MOF-303 displayed the most pronounced acceleration, achieving saturation within ∼0.3 min at 700 Pa and ∼0.1 min at 2000 Pa (Figure 4b). This rapid response reflects the high hydrophilicity and strong hydrogen bonding capability of amino functional group,^26^ which facilitates faster nucleation and propagation of water clusters within the pores. Cu–NO_2_@MOF-303 showed relatively improvements, reaching saturation in under 7 min at 700 Pa and below 3 min at 2000 Pa (Figure 4c). These results align with the observed isotherm trends, where the –NH_2_ group not only increases total uptake but also accelerates adsorption by promoting stronger initial water–framework interactions. Conversely, while –NO_2_ enhances hydrophilicity, its weaker hydrogen bonding leads to comparatively slower kinetics.

Figure 5 illustrates the temporal evolution of the real-space correction function C­(*r*), which captures changes in the local water density around adsorption sites within pristine and functionalized MOF-303 frameworks (site A, highlighted in Figure 2a). This function is calculated based on the difference between the local density of water molecules at a given radial distance and the system’s average reference density, providing insights into water density accumulation or depletion near active sites. At early stages, all systems exhibit negative C­(*r*) values at short distances, indicating initial depletion zones around the framework due to the absence of water. As adsorption proceeds, C­(*r*) shifts toward positive values, reflecting the buildup of water near active sites. Among the studied structures, Cu–NH_2_@MOF-303 shows the fastest and strongest increase in C­(*r*), indicating superior water affinity and rapid adsorption dynamics (Figure 5b). Cu–NO_2_@MOF-303 shows moderate behavior (Figure 5c), while pristine MOF-303 displays the weakest response (Figure 5a).

**Figure 5:**
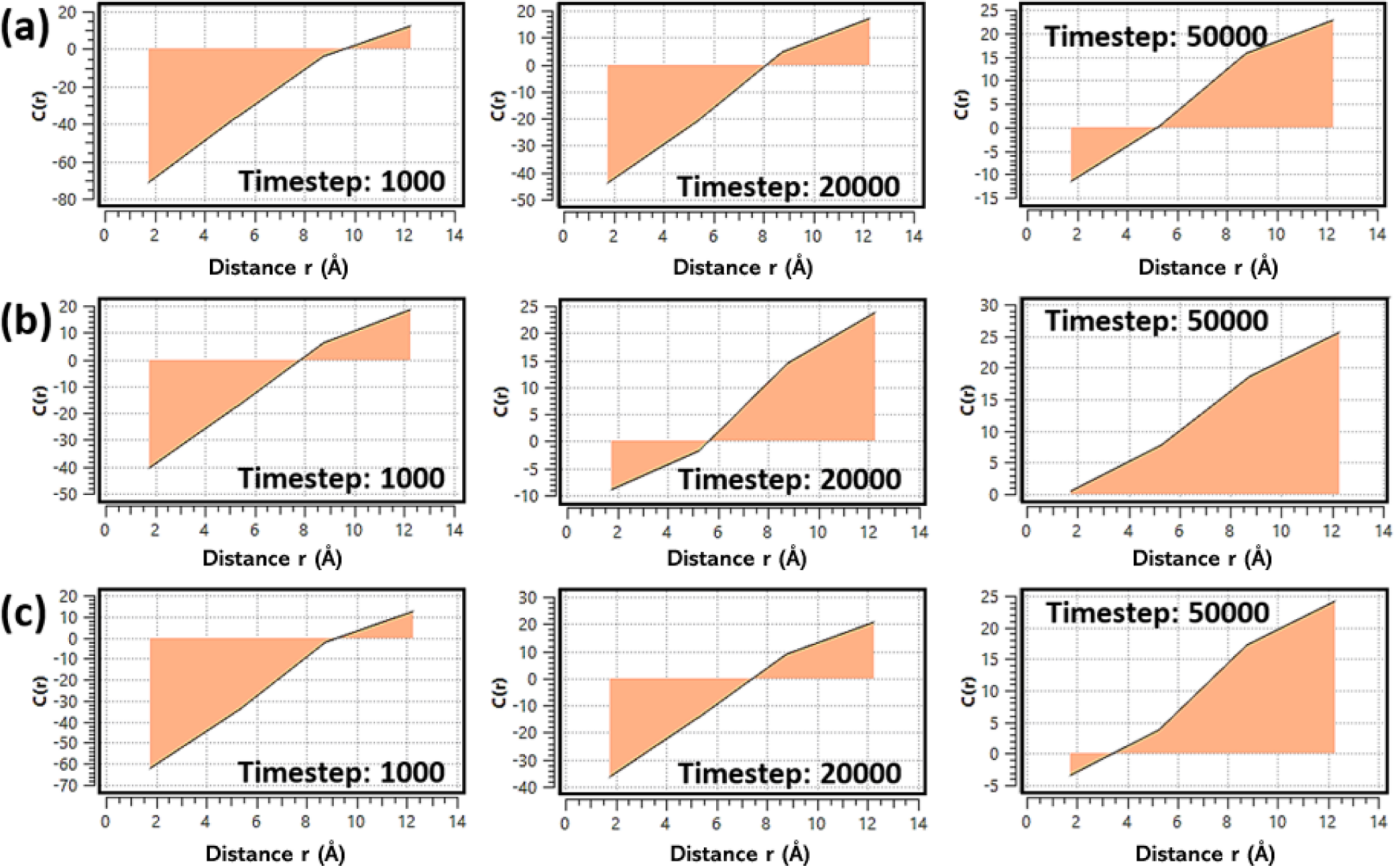
Time evolution of the real-space correction function, C­(*r*), for (a) pristine MOF-303, (b) Cu–NH_2_@MOF-303, and (c) Cu–NO_2_@MOF-303.

These observations are consistent with the kinetic adsorption results shown in Figure 3, confirming that functionalization, particularly with –NH_2_, enhances both the rate and extent of local water structuring, an important advantage for AWH under low-humidity conditions.

### 3.2 Structural and Electronic Characteristics

To gain deeper insight into the mechanism of water adsorption, we complemented the GCMC-derived isotherms and KMC-based kinetic simulations with quantum mechanical calculations using DFT. As an initial step, several configurations of water molecules were positioned on the framework models shown in Figure 2. Regardless of their starting locations, the water molecules consistently converged toward the same adsorption region, labeled as site “A” in Figure 1. Table 2 summarizes the computed adsorption energies (*E*_ads_) for pristine MOF-303 and its functionalized forms, Cu–NH_2_@MOF-303 and Cu–NO_2_@MOF-303. The pristine MOF-303 exhibited an *E*_ads_ of −84.77 kJ mol^–1^, which aligns reasonably well with values reported in prior DFT studies,^10^ with differences attributable to variations in computational protocols and differences in molecular structure used. In particular, the energies reported in ref 10 correspond to periodic DFT binding energies averaged over multiple water molecules and include cumulative framework-water and water–water interactions, whereas the values obtained in this study represent site-specific adsorption energies computed from a cluster model. Thus, the two data sets reflect different physical quantities, explaining their numerical differences while remaining fully consistent in identifying the same strongest adsorption region. This agreement supports the validity of our DFT approach. The inclusion of Cu–X groups (X = NH_2_, NO_2_) was found to enhance the interaction strength with water. Among the functionalized systems, Cu–NH_2_@MOF-303 exhibited the most negative adsorption energy (−99.93 kJ mol^–1^), followed by Cu–NO_2_@MOF-303 (−90.16 kJ mol^–1^), indicating improved water affinity upon functionalization. These findings suggest that ligand substitution modifies the host–guest interaction strength, likely through changes in the local electronic environment and polarization behavior of the framework. The high adsorption energy of Cu–NH_2_@MOF-303 is consistent with its superior water uptake observed in GCMC simulations and its rapid adsorption kinetics. Although Cu–NO_2_@MOF-303 exhibits a slightly stronger adsorption energy than the pristine structure, its lower uptake at low pressure (Figure 3b) likely results from a reduction in accessible pore volume or suboptimal initial nucleation conditions for water clustering,^26^ despite its stronger binding sites. This finding also supports the slightly enhanced adsorption kinetics observed for Cu–NO_2_@MOF-303 in Figure 4c, as the moderately stronger adsorption energy compared to pristine MOF-303 provides a plausible explanation for its marginally faster water uptake. Overall, the faster adsorption dynamics observed for Cu–NO_2_@MOF-303, and more notably for Cu–NH_2_@MOF-303, are consistent with their more favorable adsorption energies. To further explore the electronic origin of these trends, we examined several quantum chemical descriptors. Specifically, charge distribution analyses, molecular electrostatic potential (MEP) mapping, density of state (DOS) and Frontier molecular orbital characteristics (HOMO–LUMO), and finally dipole moment (μ), were considered to provide a more complete picture of the electronic structure and adsorption behavior.

**Table 2:** Calculated Water Adsorption Energies in Pristine and Cu–X@MOF-303 (X = NH_2_, NO_2_) Frameworks

adsorbent	*E*_ads_ (kJ mol^–1^)	ref.
MOF-303	–71.86	^10^
MOF-303	–84.77	this study
Cu–NH_2_@MOF-303	–99.93	this study
Cu–NO_2_@MOF-303	–90.16	this study

To gain a deeper understanding of the electronic influence exerted by amino and nitro group substitution on the Cu adsorption site in MOF-303, NBO and Mulliken charge analyses were carried out and illustrated in Figure 6. Charge distribution analysis via both Mulliken and NBO methods reveals that in Cu–NH_2_@MOF-303, the nitrogen atom carries a substantially negative charge (NBO: −0.991; Mulliken: −0.712), while the two hydrogen atoms exhibit significant positive charges, indicative of a strong ability to form directional hydrogen bonds with water molecules. In contrast, the nitrogen atom in Cu–NO_2_@MOF-303 is positively charged (NBO: +0.22; Mulliken: +0.026), and the oxygen atoms, although moderately negative, display lower electron density compared to the NH_2_ nitrogen. To further clarify the differences between the two methods, it is noted that Mulliken charges, although computationally simple, are highly sensitive to the choice of basis set and molecular geometry, and their partition electron density equally between bonded atoms regardless of electronegativity.^73^ In contrast, NBO charges are derived from natural atomic orbitals and provide a more physically meaningful description of electron distribution, reflecting bond covalency and polarization more accurately.^74^ Therefore, while both methods offer qualitative insights, NBO charges are generally considered closer to the actual electronic environment, particularly for understanding local interactions such as those between functional groups and adsorbed water molecules. Generally, these findings explain the superior adsorption energy and faster water uptake observed for Cu–NH_2_@MOF-303. The results confirm that electronic charge distribution plays a pivotal role in dictating water affinity.

**Figure 6:**
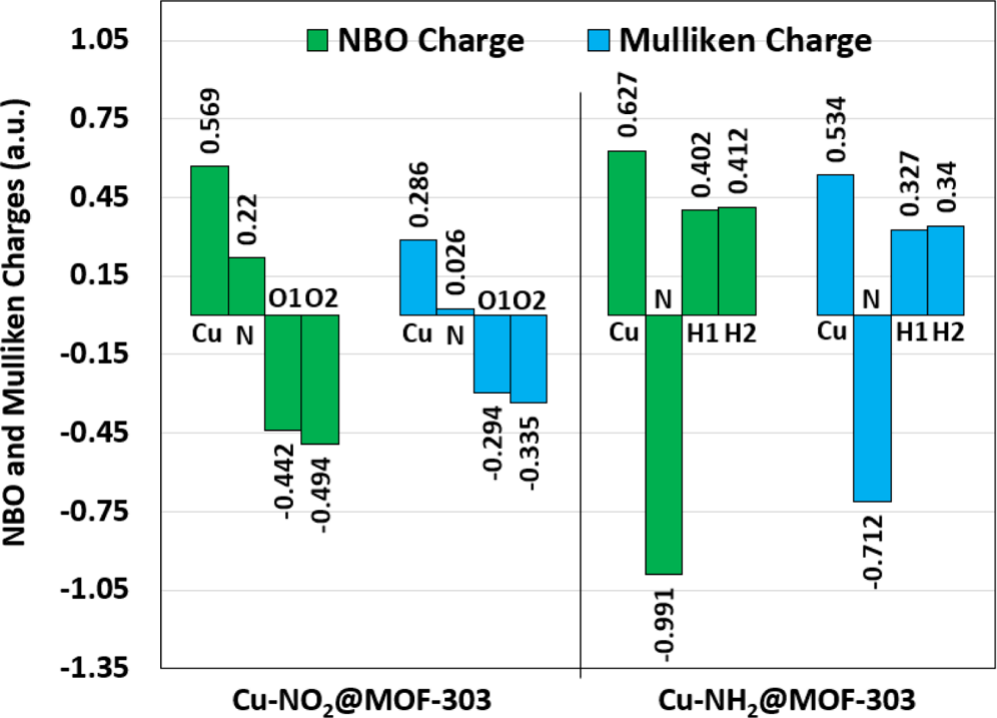
Atomic charge distributions of Cu–NH_2_@MOF-303 and Cu–NO_2_@MOF-303 calculated via Mulliken and NBO analyses, highlighting electronic differences induced by functional group substitution.

The MEP analysis was employed to gain deeper insight into the three-dimensional electronic and topological characteristics of the compounds under study. This method enables a visual interpretation of how electron density is distributed across the molecular surface, offering clues about regions that are more likely to participate in electrophilic or nucleophilic interactions. While atomic charges from NBO and Mulliken highlight localized electronic effects, the MEP surfaces offer a broader perspective on how these effects manifest across the molecular topology. The MEP surface is depicted using a continuous color scale, where blue areas signify regions of electrostatic deficiency (positive potential) and red areas highlight regions of electron accumulation (negative potential). Traditionally, red zones are indicative of sites with elevated electron density, which tend to interact favorably with electrophilic species.^75^ Figure 7a–c illustrates the MEP profiles for MOF-303 in both its unmodified form and after metalation with Cu–NH_2_ and Cu–NO_2_. In the pristine framework (Figure 7a), the potential surface appears relatively uniform and symmetric. Slightly negative regions are observed in the cavity between ligands. Meanwhile, the ligand’s aromatic rings display mostly neutral or weakly negative potential (yellow hues). Upon incorporation of Cu–NH_2_ and Cu–NO_2_ (Figure 7b,c), the MEP surface undergoes alteration. As seen in the MEP profiles, the NO_2_-functionalized framework displays pronounced red regions around the oxygen atoms, indicating strong localized electron density, which could contribute to enhanced water-framework interactions at these sites. In contrast, despite the highly negative partial charge on the nitrogen atom in the NH_2_ group, the corresponding MEP surface shows only faint yellow or even near-neutral (green) regions around the amino moiety. This apparent discrepancy is attributed to the spatial orientation and delocalization of the lone pair on the NH_2_ nitrogen, which may not project significantly into the pore space or toward the surface, thus reducing the observed surface potential in the MEP map. Consequently, while NO_2_ offers more prominent electrostatic attraction sites on the surface, the NH_2_ group still facilitates stronger water interactions through hydrogen bonding-arising from directional lone-pair donation-explaining its superior water uptake performance observed in GCMC and KMC results. This finding underscores that relying solely on MEP maps can be misleading when evaluating adsorption performance, as they reflect only the electrostatic surface potential and may overlook critical aspects such as localized charge distribution. A more accurate and holistic interpretation is achieved by integrating MEP results with partial charge analysis that offering a multifaceted perspective that links electronic structure with actual adsorption behavior.

**Figure 7:**
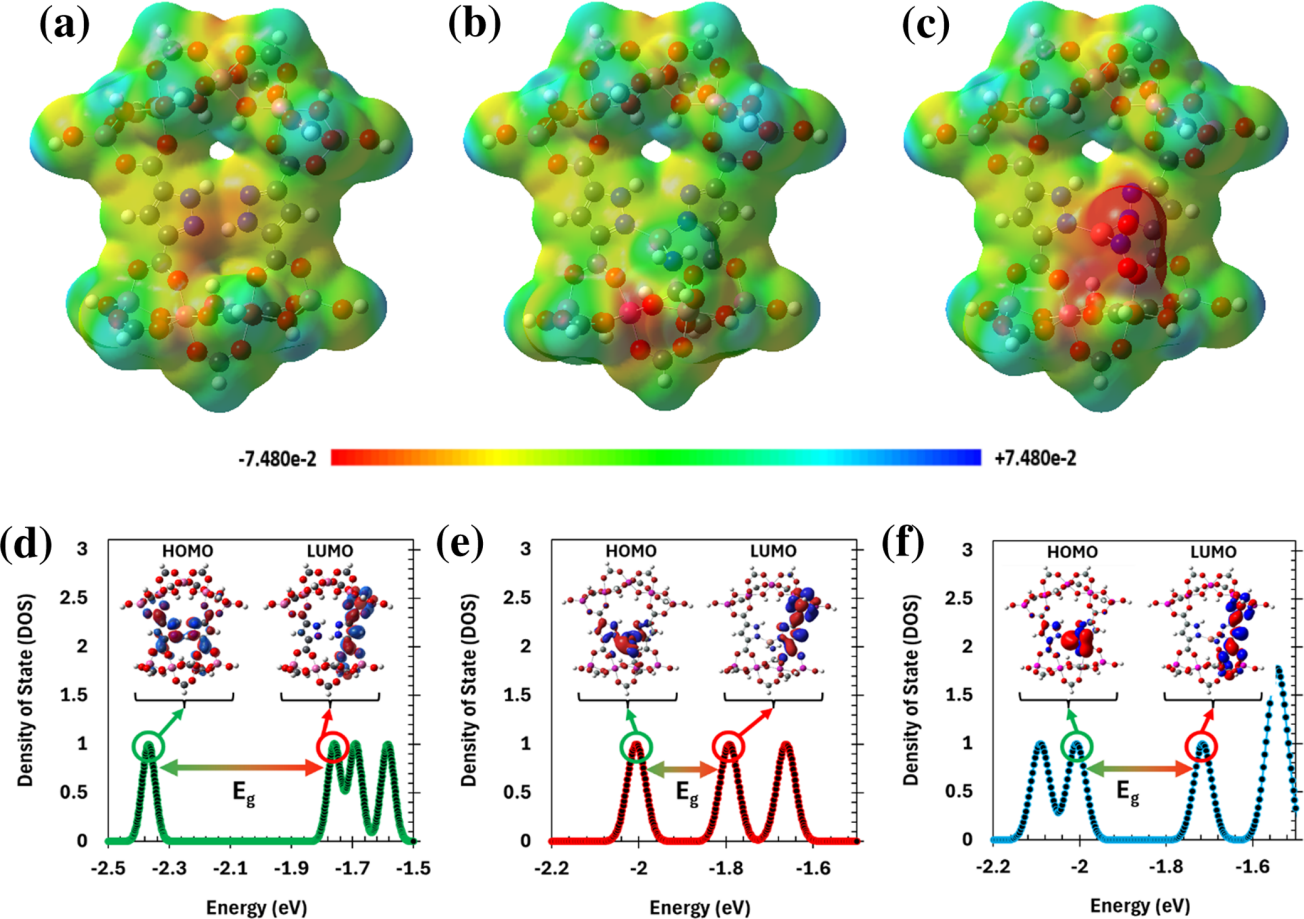
(a–c) MEP maps for (a) pristine MOF-303, (b) Cu–NH_2_@MOF-303, and (c) Cu–NO_2_@MOF-303. Red regions indicate areas of high electron density (negative potential), while blue regions represent electron-deficient zones (positive potential). (d–f) DOS plots and corresponding visualizations of the HOMO and LUMO orbitals for (d) pristine MOF-303, (e) Cu–NH_2_@MOF-303, and (f) Cu–NO_2_@MOF-303. Functionalization reduces the HOMO–LUMO energy gap and alters Frontier orbital delocalization, particularly for Cu–NH_2_, indicating enhanced electronic reactivity and adsorption potential.

To further investigate the electronic effects induced by functional group substitution, DOS analyses were performed for pristine and functionalized MOF-303 frameworks. Each DOS plot is presented alongside the corresponding HOMO and LUMO orbital visualizations (Figure 7d–f), facilitating a direct comparison between the energy distribution and spatial localization of Frontier orbitals. As shown, both Cu–NH_2_ (Figure 7e) and Cu–NO_2_ (Figure 7f) functionalization result in a noticeable narrowing of the HOMO–LUMO energy gap compared to the pristine framework, which aligns with the numerical values previously discussed. This reduced gap suggests enhanced electronic polarizability and reactivity, features that enhance water interactions and charge redistribution during adsorption. The effect is particularly pronounced for Cu–NH_2_@MOF-303, where the energy gap contraction is more significant, and the Frontier orbitals exhibit a highly delocalized and asymmetric distribution across the framework. Such spatial delocalization implies greater orbital overlap and facilitates stronger and faster interactions with incoming water molecules, corroborating the observed trends in adsorption energy (Table 2), GCMC uptake (Figure 3), and kinetic enhancement (Figure 4). Additionally, the increased asymmetry and spread of HOMO and LUMO orbitals in the functionalized structures indicate modified charge transport pathways and a more flexible electronic environment for accommodating adsorbates. These findings further reinforce the conclusion that NH_2_ functionalization introduces not only stronger binding affinity but also greater adaptability at the electronic level, both of which are crucial for efficient water adsorption under low-pressure conditions.

Table 3 summarizes the calculated dipole moments (μ) for pristine MOF-303 and its functionalized counterparts, Cu–NH_2_@MOF-303 and Cu–NO_2_@MOF-303, before and after water adsorption. A systematic enhancement in the intrinsic μ is observed upon functionalization, reflecting significant alterations in the electronic asymmetry and charge polarization of the framework. Specifically, the pristine MOF-303 exhibits a relatively low dipole moment of 2.137 D, which increases markedly to 6.182 D for Cu–NH_2_@MOF-303 and further to 8.478 D for Cu–NO_2_@MOF-303. This trend indicates that both functional groups introduce substantial polarity, with NO_2_ imparting the highest intrinsic dipolar character.

**Table 3:** Computed Dipole Moments (μ) of Pristine MOF-303 and Cu–X@MOF-303 (X = NH_2_, NO_2_) Frameworks before and after Water Adsorption, along with the Corresponding Change (Δμ)

adsorbent	μ before adsorption (D)	μ after adsorption (D)	|Δμ| (D)
MOF-303	2.137	3.682	1.545
Cu–NH_2_@MOF-303	6.182	10.044	3.862
Cu–NO_2_@MOF-303	8.478	5.967	2.511

Upon water adsorption, however, the change in dipole moment (Δμ) exhibits a distinct trend. Cu–NH_2_@MOF-303 shows the largest increase in dipole moment (Δμ = +3.862 D), followed by Cu–NO_2_@MOF-303 (Δμ = −2.511 D, negative due to reduction), and pristine MOF-303 (Δμ = +1.545 D). The substantial increase in Δμ for Cu–NH_2_@MOF-303 suggests a strong polarization response induced by water, likely mediated through directional hydrogen bonding between the lone pair of the amino nitrogen and incoming water molecules. This behavior aligns with previous findings, where the NH_2_-functionalized framework exhibited the highest adsorption energy and fastest kinetics. The water-induced dipole enhancement reflects efficient charge rearrangement and the development of localized electric fields favorable for nucleation and cluster growth.

In contrast, despite its higher intrinsic μ, Cu–NO_2_@MOF-303 experiences a decrease in μ after water adsorption. This counterintuitive behavior can be explained by considering the electron-withdrawing nature of the NO_2_ group,^76^ which may rigidify the local electrostatic field and reduce its adaptability upon guest interaction. Additionally, as visualized in the MEP maps (Figure 7c), the highly localized electron density around the oxygen atoms of NO_2_ creates fixed interaction zones, leading to more constrained water binding and lower configurational freedom, thereby diminishing the overall dipole response.

The relatively small Δμ observed for pristine MOF-303 reflects its symmetric and less polar framework, which undergoes limited electronic reorganization upon adsorption. Together, these results demonstrate that Δμ serves as an effective indicator of adsorption-induced polarization, where stronger Δμ correlates with enhanced water–framework interaction dynamics. The mismatch between the intrinsic dipole moment and the adsorption-induced dipole variation (particularly for Cu–NO_2_@MOF-303) underscores the limitations of using static electronic properties alone to predict adsorption performance. Rather, a comprehensive understanding necessitates integrating dynamic electronic responses, such as Δμ, with charge distribution (NBO/Mulliken), MEP surface characteristics, and orbital analyses (DOS, HOMO–LUMO) to fully capture the adsorption behavior and reactivity of functionalized MOFs.

### 3.3 Molecular-Level Study

To further elucidate the local interaction environments and spatial characteristics of water adsorption within functionalized MOF-303 frameworks, RDF analyses were carried out for key atomic pairs involved in water coordination. Specifically, RDFs were computed between copper and the oxygen atom of water molecules (Cu–O­(W)), and between nitrogen in the amino/nitro group and the hydrogen of water (N–H­(W)), across two RH levels (RH = 8% and 22%). These conditions were selected to probe early stage (low coverage) and intermediate-stage (moderate coverage) water adsorption regimes (Figure 8a–d).

**Figure 8:**
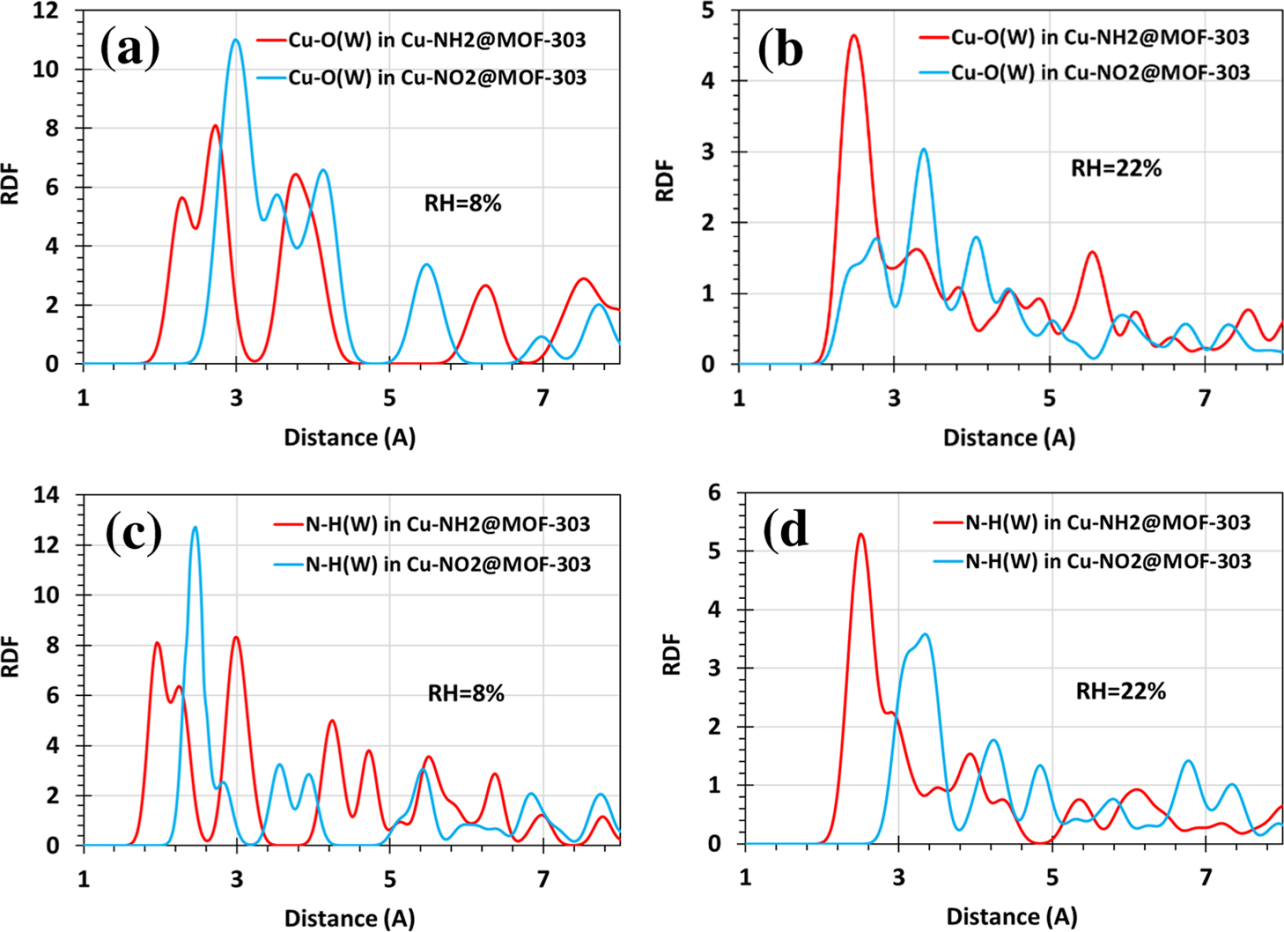
RDFs characterizing the local water–framework interactions in functionalized MOF-303 structures at two relative humidity levels (RH = 8% and 22%) at 300 K. (a,b) RDFs of Cu–O­(water) pairs in Cu–NH_2_@MOF-303 and Cu–NO_2_@MOF-303, showing closer coordination in the NH_2_-functionalized system. (c,d) RDFs of N–H­(water) in Cu–NH_2_@MOF-303 and Cu–NO_2_@MOF-303, highlighting stronger and more directional hydrogen bonding in the amino-functionalized structure.

Figure 8a,b illustrate the RDF profiles for the Cu–O­(W) interactions in Cu–NH_2_@MOF-303 and Cu–NO_2_@MOF-303 at RH = 8% and 22%, respectively. At both humidity levels, distinct peaks are observed in the range of ∼2.2–2.6 Å, consistent with coordination between the water oxygen and the Cu center. Notably, at RH = 8% (Figure 8a), Cu–NH_2_@MOF-303 exhibits an RDF peak at a slightly shorter distance compared to Cu–NO_2_@MOF-303. This behavior indicates a stronger electrostatic attraction between the Cu site and water in the amino-functionalized framework, which can be attributed to the higher positive partial charge on Cu as a result of NH_2_ substitution, as confirmed by Mulliken and NBO analyses (Figure 6). The closer proximity of water molecules to Cu in Cu–NH_2_@MOF-303 facilitates earlier nucleation events and enhances site-specific adsorption under dry conditions. At RH = 22% (Figure 8b), both systems display RDF peaks with reduced intensity, indicating a partial delocalization of water molecules due to the formation of water–water hydrogen bonds and a decreased dependency on specific adsorption sites. Nevertheless, Cu–NH_2_@MOF-303 continues to exhibit a slightly closer and more defined peak compared to Cu–NO_2_@MOF-303, suggesting that its Cu centers retain stronger interactions with water even at higher loading.

Figure 8c,d present RDFs for N–H­(W) in Cu–NH_2_@MOF-303 and Cu–O­(W) in Cu–NO_2_@MOF-303 at RH = 8% and 22%, respectively. In Figure 8c (RH = 8%), the RDF for N–H­(W) in Cu–NH_2_@MOF-303 exhibits a well-defined peak at ∼1.8–2.0 Å, characteristic of directional hydrogen bonding. This result confirms the crucial role of the –NH_2_ functional group in acting as a hydrogen bond donor, thereby enabling strong and localized interactions with water molecules during the initial adsorption stages. In contrast, the Cu–O­(W) RDF in Cu–NO_2_@MOF-303 shows a more distant peak, indicating weaker or less directional interactions. At RH = 22% (Figure 8d), the intensity of the N–H­(W) peak slightly diminishes, reflecting increasing water–water interactions; however, its presence at a consistently short distance underscores the persistent hydrogen bonding capability of the –NH_2_ group even at moderate coverage. These RDF trends are consistent with the more negative water adsorption energy of −99.93 kJ mol^–1^ (Table 2), faster adsorption kinetics (Figure 4b), and greater low-pressure uptake (Figure 3b) observed for the amino-functionalized structure. Conversely, the nito-functionalized structure consistently exhibits more distant RDF peaks, which correspond to its weaker adsorption performance across all other analyses. This discrepancy highlights the limited adaptability of the NO_2_ group in forming strong and flexible interactions with water molecules, likely due to its electron-withdrawing character and spatially constrained electrostatic fields (as shown in MEP maps, Figure 7c).

Overall, RDF analysis confirms that –NH_2_ functionalization introduces stronger, more directional, and shorter interactions with water, both via electrostatic Cu–O coordination and hydrogen bonding through the NH_2_ moiety. These interactions are crucial in driving the superior water uptake behavior, particularly under low RH conditions, making Cu–NH_2_@MOF-303 a highly promising candidate for efficient atmospheric water harvesting applications.

To further explore the dynamic behavior of water molecules within the MOF-303 frameworks, MSD analyses were performed at RH = 22% and *T* = 300 K for the pristine and functionalized systems: MOF-303, Cu–NO_2_@MOF-303, and Cu–NH_2_@MOF-303 (Figure 9a). The MSD curves reveal clear differences in the translational mobility of water molecules across the three structures. Pristine MOF-303 exhibits the highest MSD slope, reflecting the highest degree of molecular motion due to weaker water–framework interactions. In contrast, Cu–NH_2_@MOF-303 shows the most subdued MSD profile, indicating more restricted mobility resulting from stronger water-framework binding. Cu–NO_2_@MOF-303 lies in between, suggesting an intermediate level of water confinement.

**Figure 9:**
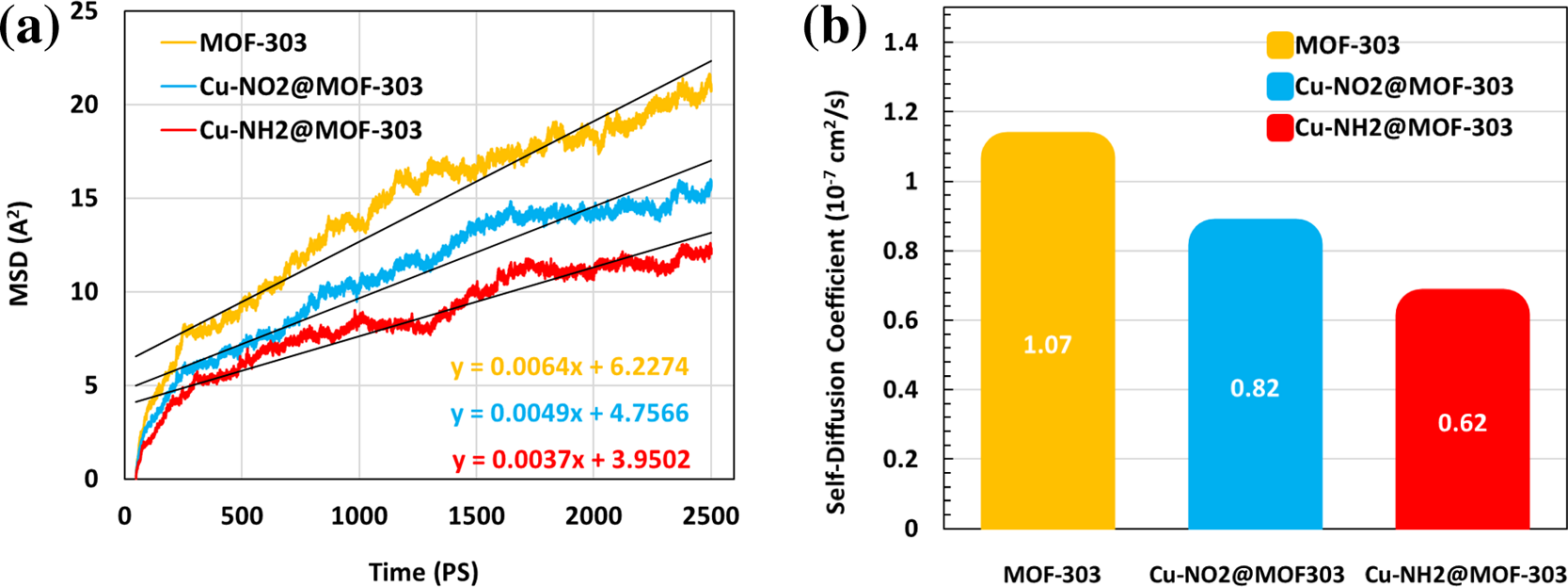
MSD profiles and diffusion coefficients of water molecules in pristine and functionalized MOF-303 frameworks at RH = 22% and *T* = 300 K. (a) MSD curves showing differences in water translational mobility across MOF-303, Cu–NO_2_@MOF-303, and Cu–NH_2_@MOF-303. (b) Calculated diffusion coefficients extracted from the linear region of MSD profiles, highlighting the inverse relationship between water mobility and adsorption strength.

These trends are consistent with previous observations of adsorption energy and water uptake. The amino-functionalized framework, which displayed the most negative water adsorption energy (−99.93 kJ mol^–1^), also shows the most restricted water mobility—a direct consequence of strong hydrogen bonding and electrostatic interactions that hinder translational motion. Conversely, in the pristine MOF-303, where adsorption interactions are weaker (*E*_ads_ = −84.77 kJ mol^–1^), water molecules are less confined and thus diffuse more freely.

To quantitatively evaluate water mobility, diffusion coefficients (*D*) were extracted from the linear region of each MSD curve using the Einstein relation (Figure 9b). The results follow the same order observed in MSD profiles: MOF-303 exhibits the highest diffusion coefficient, followed by Cu–NO_2_@MOF-303, and finally Cu–NH_2_@MOF-303 with the lowest value. This inverse relationship between diffusion coefficient and adsorption strength further validates the previous thermodynamic and structural findings—materials with stronger water–framework interactions tend to exhibit reduced water mobility due to stronger binding and spatial confinement.^77^

Altogether, the MSD and diffusion data reinforce the conclusions drawn from RDF, adsorption energy, and kinetic analyses. In particular, the significantly reduced diffusivity observed in Cu–NH_2_@MOF-303 highlights its strong water retention capability, which is desirable for water harvesting under dry conditions where sustained adsorption is preferred over rapid desorption.

### 3.4 Desorption Study

Understanding the thermal desorption behavior of water molecules is essential for evaluating the practical performance and regeneration feasibility of MOF-based materials in AWH applications. Efficient materials must not only exhibit strong water uptake under low humidity but also enable energy-efficient desorption at moderate temperatures. If desorption requires excessively high thermal input, the system’s operational viability and sustainability are significantly compromised.

To evaluate the thermal response of water–framework interactions, temperature-dependent adsorption energy fluctuations were monitored for pristine MOF-303, Cu–NO_2_@MOF-303, and Cu–NH_2_@MOF-303 across a temperature range of 100–800 K (Figure 10a–c). In all three systems, increasing temperature results in enhanced fluctuations of adsorption energy, reflecting reduced thermodynamic stability and more dynamic water binding at higher thermal energy. Notably, the amplitude of fluctuations is greater in the functionalized frameworks compared to the pristine structure, with Cu–NO_2_@MOF-303 exhibiting the largest variation. This behavior indicates that functional groups such as –NO_2_ and –NH_2_ introduce more thermally sensitive interactions.

**Figure 10:**
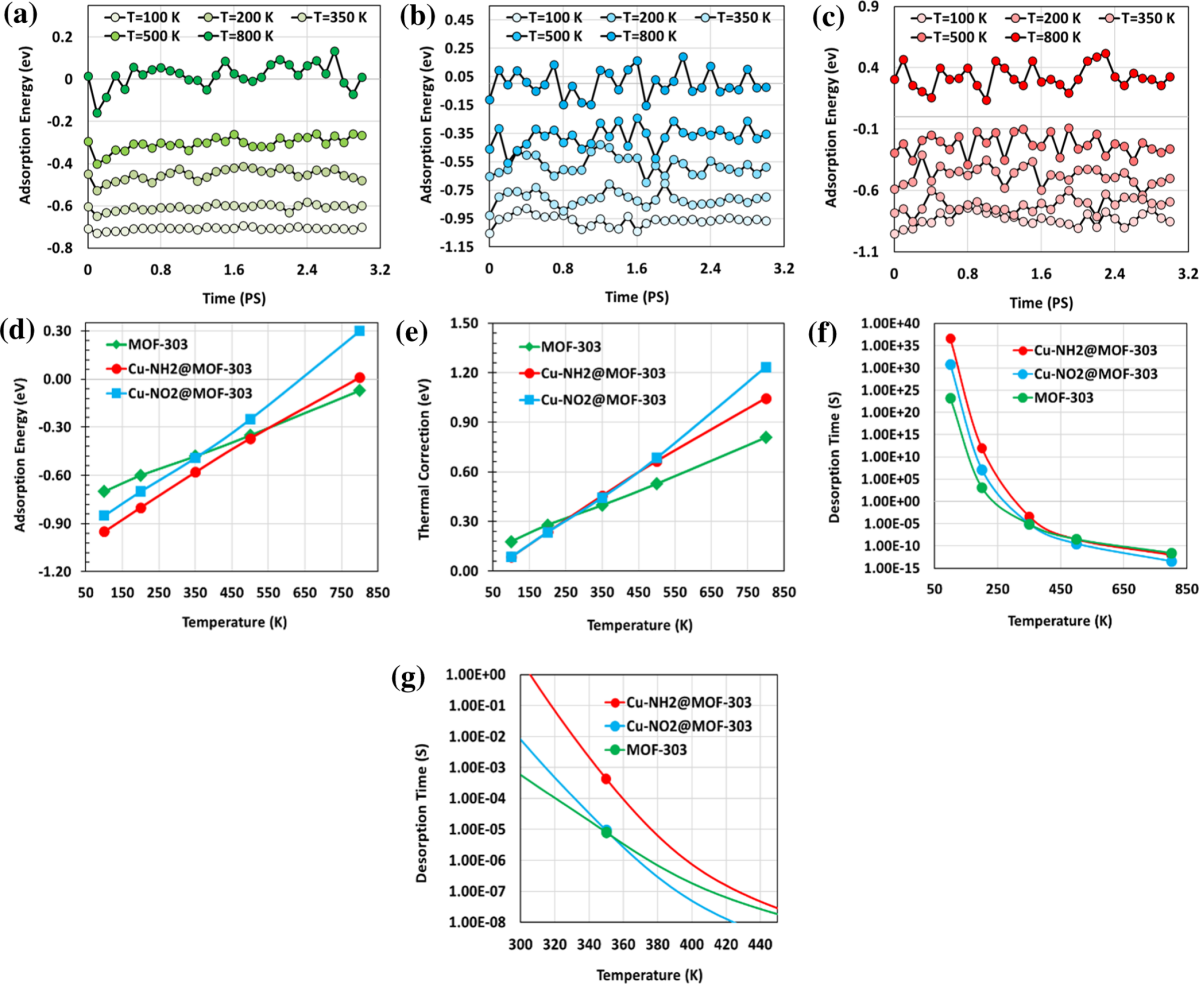
(a–c) Time-dependent fluctuations in adsorption energies at various temperatures (100–800 K) for MOF-303, Cu–NO_2_@MOF-303, and Cu–NH_2_@MOF-303, respectively. (d) Average adsorption energies of H_2_O across the same temperature range for all three structures. (e) Corresponding thermal corrections of adsorption energies. (f) Estimated temperature-dependent desorption time constant (τ) for pristine MOF-303 and Cu–X@MOF-303 (X: NO_2_, NH_2_) over the full range of 100–800 K and (g) focused view between 300 and 450 K.

Figure 10d shows the average adsorption energies of water at different temperatures. A consistent trend of decreasing adsorption strength with increasing temperature is observed across all systems. Among them, Cu–NO_2_@MOF-303 displays the steepest decline in adsorption energy, followed by Cu–NH_2_@MOF-303, while pristine MOF-303 shows the least temperature sensitivity. This trend suggests that although functional groups enhance binding affinity at lower temperatures, they are more susceptible to thermal weakening. This observation is further supported by the thermal correction values (Figure 10e), which increase with temperature in all cases but do so more significantly in the functionalized structures. Cu–NO_2_@MOF-303 exhibits the largest thermal correction, followed by Cu–NH_2_@MOF-303 and pristine MOF-303, reflecting the greater entropic contributions and configurational flexibility introduced by polar functional groups.

To quantify the desorption kinetics, temperature-dependent desorption time constants (τ) were calculated based on adsorption energies (Figure 10f). As expected, τ values decrease exponentially with increasing temperature, indicating accelerated water release at higher thermal input. In the operational temperature range relevant to AWH systems (approximately 300–400 K), the τ values offer important insights into regeneration performance. At 350 K, pristine MOF-303 exhibits a characteristic desorption time of 8.1 × 10^–6^ s, which serves as a reference for comparison. The nitro-functionalized structure demonstrates τ values of the same order of magnitude at this temperature, suggesting that water can be effectively released under practical operating conditions despite stronger adsorption. However, to match the desorption performance of pristine MOF-303 at 350 K, Cu–NH_2_@MOF-303 requires a temperature of approximately 375 K. This small increase in regeneration temperature is a direct consequence of the stronger initial binding strength introduced by amino functionalization and is in line with the higher adsorption energies and lower diffusivities previously reported. Despite this slight thermal penalty, both functionalized MOFs retain practical regeneration capability and reversible adsorption/desorption behavior.

To quantitatively assess the regeneration cost and provide a practical performance metric for SAWH applications, the specific energy consumption (SEC) and coefficient of performance (COP) were estimated for pristine MOF-303 and the two functionalized structures. A constant desorption rate was adopted as the reference criterion, reflecting the characteristic desorption behavior of pristine MOF-303 at 350 K. From the desorption time-constant analysis, the regeneration temperatures required to achieve the same desorption rate were estimated to be approximately 350 K for MOF-303 and Cu–NO_2_@MOF-303, and 375 K for Cu–NH_2_@MOF-303. The SEC indicator represents the specific energy consumption related to water production.^78^ Although the exact SEC calculation would require accounting for all thermal contributions, including the latent heat of adsorption and the sensible heat required to heat the sorbent, vapor, and air;^79–83^ here, for the sake of a comparative assessment, we neglected the sensible heat and considered only the latent heat, which corresponds to the adsorption enthalpy^83^ and is approximately equivalent to the temperature-dependent adsorption energies obtained from thermal corrections at these regeneration temperatures.^84,85^ Therefore, we approximated SEC by normalizing the temperature-dependent adsorption energies at these regeneration temperatures (MOF-303: ∼−0.48 eV, Cu–NO_2_@MOF-303: ∼−0.48 eV, Cu–NH_2_@MOF-303: ∼−0.55 eV) to the mass of water (SEC ≈ |*E*_ads_(*T*)|/M_H_2_O_). The COP was then calculated as the ratio of the latent heat of water vaporization (*L*_v_) to this approximate SEC value,^86,87^ where *L*_v_ represents the enthalpy of vaporization in the relevant operational temperature range (COP = *L*_v_/SEC, with *L*_v_ ≈ 2.316 MJ kg^–1^ (at 350 K) and 2.251 MJ kg^–1^ (at 375 K)). This approximation allows for a meaningful comparison of materials under equivalent desorption conditions, despite the simplifications involved. The results, summarized in Table 4, show that Cu–NO_2_@MOF-303 exhibits a regeneration energy cost nearly identical to pristine MOF-303, reflecting its comparable adsorption strength at 350 K. In contrast, Cu–NH_2_@MOF-303 requires a moderately higher regeneration temperature and exhibits a higher SEC (2.948 MJ kg^–1^), corresponding to its stronger water binding. Despite this modest penalty, the SEC values for all three materials confirming that both functionalized MOFs maintain practical regeneration feasibility.

**Table 4:** SEC and COP for Pristine and Functionalized MOF-303 Materials under Equal Desorption-Rate Conditions Referenced to Pristine MOF-303

adsorbents	regeneration temperature (K)	adsorption energy (eV)	SEC (MJ kg^–1^)	COP (−)
MOF-303	350	≈−0.48	≈2.571	≈0.901
Cu–NH_2_@MOF-303	≈375	≈−0.55	≈2.948	≈0.763
Cu–NO_2_@MOF-303	≈350	≈−0.48	≈2.571	≈0.901

Overall, Cu–NH_2_@MOF-303, despite requiring a regeneration temperature approximately 25 K higher than pristine MOF-303, offers a favorable trade-off between enhanced water uptake and an acceptable increase in desorption energy, making it well-suited for low-to-moderate RH operating conditions where higher sorption capacity is valuable. In contrast, Cu–NO_2_@MOF-303 matches the regeneration behavior of pristine MOF-303 while maintaining competitive saturated adsorption performance at high RH, suggesting that it may be the more practical choice under very high-humidity environments where rapid and energy-efficient water release is critical. This balance between adsorption strength and regeneration efficiency underscores the potential of functionalized MOF-303 materials as effective candidates for real-world SAWH applications.

## 4 Conclusions and Future Outlook

This study systematically investigated the effect of functional group substitution on the water adsorption performance of MOF-303 for AWH, employing a multiscale computational framework combining GCMC, KMC, MD, DFT, and temperature-dependent desorption analysis. Functionalization with Cu–NH_2_ and Cu–NO_2_ groups significantly altered the framework’s structural and electronic environment, enhancing water affinity and modifying adsorption dynamics. Cu–NH_2_@MOF-303 exhibited the highest adsorption energy (−99.93 kJ mol^–1^), rapid uptake kinetics, strong hydrogen bonding, and reduced water diffusivity-making it highly effective across all humidity ranges. Although it required a moderately higher regeneration temperature (∼375 K), this remains within practical operational limits. In contrast, Cu–NO_2_@MOF-303 offered easier desorption behavior, with regeneration dynamics comparable to pristine MOF-303, making it suitable for high-humidity applications where energy-efficient release is crucial. Electronic structure analyses, including charge distribution, MEP mapping, DOS, and dipole response, provided mechanistic insight into the nanoscale host–guest interactions and adsorption-induced polarization. RDF and MSD analyses further confirmed localized binding and mobility trends consistent with adsorption performance.

Overall, this work highlights the tunability of MOF-303 through targeted nanoscale functionalization, demonstrating how the balance between strong water uptake and practical regeneration can be optimized. These insights establish Cu–NH_2_@MOF-303 as a promising candidate for AWH in arid environments, while Cu–NO_2_@MOF-303 may be preferable for humid regions with lower energy input constraints.

Beyond the molecular-level insights obtained in this work, translating these findings into practical AWH systems requires consideration of several engineering challenges. For instance, long-term cyclic stability under repeated adsorption–desorption cycling can be influenced by functional group durability, potential hydrolysis, or structural fatigue, factors that cannot be fully captured by molecular simulations alone. Likewise, scalability and manufacturing cost present practical constraints, as functionalization routes such as Cu–X docking must be experimentally optimized to ensure synthesis reproducibility, material homogeneity, and economic feasibility at large scale. While these considerations lie beyond the scope of the present computational study, the results reported here provide clear molecular-level guidance that can help prioritize future experimental investigations into stability, cost-efficient synthesis, and scale-up strategies for AWH materials.

## Supplementary Material



## Notes

Supplementary equations (S1–S9), Lennard-Jones and Coulombic interaction expressions, RDF and MSD formulations, diffusion coefficient calculation, and translational, rotational, and vibrational energy contributions (PDF)
