# Supplementary material for: Computational Prediction of the Complete Adsorption–Regeneration Cycle of Functionalized Metal–Organic Frameworks for Atmospheric Water Harvesting
Source: ACS Appl Nano Mater. 2026 Jan 20;9(5):2284–300. doi: 10.1021/acsanm.5c04879 (PMC12888646; doi:10.1021/acsanm.5c04879)
Supplement: Supplementary file 1 [file an5c04879_si_001.pdf]

Supporting Information

Computational Prediction of the Complete Adsorption-Regeneration Cycle of Functionalized Metal-Organic Frameworks for Atmospheric Water Harvesting

Mehrzad Arjmandi[a], *Mohamed Khayet*[a,b*]

[a]Department of Structure of Matter, Thermal Physics and Electronics, Faculty of Physics, University Complutense of Madrid, Avda. Complutense s/n, 28040 Madrid, Spain

[b]Madrid Institute for Advanced Studies of Water (IMDEA Water Institute), Avda. Punto Com N° 2, 28805 Alcalá de Henares, Madrid, Spain

*Corresponding authors: khayetm@fis.ucm.es

Supplementary Eq. S1-S3

Interatomic interactions were described using a combination of 12-6 Lennard-Jones (L-J) and Coulombic potentials[1,2]:

$$U_{unbound} = 4\varepsilon_{ij}\left[\left(\frac{\sigma_{ij}}{r_{ij}}\right)^{12} - \left(\frac{\sigma_{ij}}{r_{ij}}\right)^{6}\right] + \frac{q_i q_j}{4\pi\varepsilon_0 r_{ij}} \qquad (S1)$$

$$\varepsilon_{ij} = \sqrt{\varepsilon_i \varepsilon_j} \qquad (S2)$$

$$\sigma_{ij} = \frac{\sigma_i + \sigma_i}{2} \qquad (S3)$$

where ε_{ij} is the L-J well depth, σ_{ij} is the L-J collision diameter, r_{ij} is the distance between atoms i and j, q is the charge of each atom, and ε_0 is the permittivity of vacuum.

Supplementary Eq. S4

The RDF was calculated using the standard expression[3,4]:

$$RDF(r) = \frac{n_{r+dr}}{4\pi r^2 \rho dr} \qquad (S4)$$

where r is the distance from a reference atom, n_{r+dr} is the number of atoms in the spherical shell between r and $r + dr$, and ρ is the average atomic density.

Supplementary Eq. S5

The mean square displacement (MSD) provides a statistical measure of the average squared

distance that particles travel over time and is defined as[5,6]:

$$MSD(t) = \frac{1}{N}\left\langle \sum_{i=1}^{N} |r_i(t) - r_i(0)|^2 \right\rangle \quad (S5)$$

where N is the total number of water atoms considered, $r_i(0)$ and $r_i(t)$ denote the position vectors of atom i at the initial and later times, respectively, and the brackets represent the ensemble average.

Supplementary Eq. S6

The self-diffusion coefficient D of water molecules was obtained from the time-dependent MSD using Einstein's relation[5]:

$$D = \lim_{t\to\infty} \frac{MSD(t)}{6t} \quad (S6)$$

This coefficient quantifies the rate of molecular diffusion under confinement and was calculated separately for both frameworks at varying relative humidity (RH) levels.

Supplementary Eq. S7

Translational Contribution[7]

$$E_{trans}(T) = \frac{3}{2}\, R.T \quad (S7)$$

where R is the gas constant and T is the temperature.

Supplementary Eq. S8

Rotational Contribution[7]

$$E_{rot}(T) = \begin{cases} 3/2\ R.T, & for\ non-linear\ molecules\ (e.g.,H_2O) \\ R.T, & for\ linear\ molecules \end{cases} \qquad (S8)$$

where R is the gas constant and T is the temperature.

Supplementary Eq. S9

Vibrational Contribution[7-9]

$$E_{vib}(T) = \sum_{i=1}^{3N-6} \left[\frac{1}{2} h\vartheta_i + \frac{h\vartheta_i}{exp\left(\frac{h\vartheta_i}{K_B T}\right) - 1} \right] \qquad (S9)$$

where h is Planck's constant, K_B is the Boltzmann constant, T is the temperature, N denotes the total number of atoms in the molecule, and ϑ_i represents the vibrational frequency of the i-th normal mode obtained from DFT frequency analysis.

REFERENCES

(1) Li, Y.; Yu, J.; Li, Y.; Shen, J.; Du, M.; Zhang, X.; Zhao, H.; Pu, J. H. Nanoporous MOF-303 Performance for Atmospheric Water Harvesting in the Presence of Airborne Contaminants: GCMC and DFT Simulations. ACS Appl. Nano Mater. 2024, 7, 23850–23859.

(2) Kancharlapalli, S.; Snurr, R. Q. High-Throughput Screening of the CoRE-MOF-2019 Database for CO_2 Capture from Wet Flue Gas: A Multi-Scale Modeling Strategy. ACS Appl. Mater. Interfaces 2023, 15, 28084–28092.

(3) Jajko, G.; Gutiérrez-Sevillano, J. J.; Sławek, A.; Szufla, M.; Kozyra, P.; Matoga, D.; Makowski, W.; Calero, S. Water adsorption in ideal and defective UiO-66 structures. Microporous Mesoporous Mater. 2022, 330, 111555.

(4) Vicent-Luna, J.; Romero-Enrique, J.; Calero, S.; Anta, J. Micelle Formation in Aqueous Solutions of Room Temperature Ionic Liquids: A Molecular Dynamics Study. J. Phys. Chem. B 2017, 121, 8348–8358.

(5) Bessa, M. C.; Luna-Triguero, A.; Vicent-Luna, J. M.; Carmo, P. M.; Tsampas, M. N.; Ribeiro, A. M.; Rodrigues, A. E.; Calero, S.; Ferreira, A. F. An Efficient Strategy for Electroreduction Reactor Outlet Fractioning into Valuable Products. Ind. Eng. Chem. Res. 2023, 62, 8847–8863.

(6) Liu, Z.; Cheng, Q.; Li, K.; Wang, Y.; Zhang, J. The interaction of nanoparticulate Fe2O3 in the sintering process: A molecular dynamics simulation. Powder Technol. 2020, 367, 97-104.

(7) Aschi, M.; Grandinetti, F. The ionization potential of NF3: a G3 computational study on the thermochemical properties of NFx and NFx+ (x=1–3). J. Mol. Struct.: THEOCHEM 2000, 497, 205-209.

(8) Atkins, P.; Paula, J. d. Atkins' physical chemistry (8th ed.). Oxford University Press 2006.

(9) Sekerka, R. F. Grand Canonical Ensemble. Thermal Physics (Thermodynamics and Statistical Mechanics for Scientists and Engineers), Elsevier 2015, 359-396.
